# Influence of Cr Ion Bombardment on the Growth of Cu Coatings Deposited by Magnetron Sputtering on ABS Substrates

**DOI:** 10.3390/polym15010080

**Published:** 2022-12-25

**Authors:** Wei Dai, Zhixue Liu, Melvin Lim

**Affiliations:** 1 School of Electromechanical Engineering, Guangdong University of Technology, Guangzhou 510006, China; 2 School of Engineering, Singapore Institute of Technology, 10 Dover Drive, Singapore 138683, Singapore

**Keywords:** polymer metallization, ion bombardment, growth structure, adhesion, ABS

## Abstract

Cu coatings were deposited on acrylonitrile-butadiene-styrene copolymer (ABS) substrates by DC magnetron sputtering with Cu target. Cr ions generated by arc evaporation were used to bombard the ABS substrates before the Cu coating process. The influences of the Cr ion bombardment on the surface topography and chemical bonds of the ABS substrates and the adhesion of the Cu coatings on the ABS substrate were studied using scanning electron microscopy, Fourier transform infrared spectroscopy, and micro-Scratch Tester as a function of bias voltage and treatment duration. The results show that the Cr ion bombardment causes Cr particles to embed in the surface. The Cr particles can interlock with the Cu coatings and ABS substrate and significantly improve the coating adhesion. In addition, the Cr particles can act as the nucleation sites of the Cu coatings and facilitate the growth of columnar crystals. Increasing the duration of Cr ion bombardment increases the number of Cr particles and, thus, enhances the adhesion. However, the continuous bombardment results in the degeneration of the ABS surface, causing the formation of the coarse columnar structure of the Cu coatings. Increasing the bias voltage can increase the energy of the Cr particles without causing degeneration of the ABS. The Cu coating deposited on the ABS substrate treated by Cr ion with high-bias voltage and short duration shows a dense and smooth growth structure. In contrast, the bombardment of the Cr ions carried out at high-bias voltage induces the formation of an interfacial layer (amorphous carbon-rich phase) in the ABS surface, which decreases the coating adhesion. It is believed that Cu coatings with strong adhesion and dense structures could be acquired on ABS substrates by optimizing the bias voltage and duration of the Cr ion bombardment pre-treatment.

## 1. Introduction

Polymer metallization has attracted much research focus on its wide-ranging applications in different industries, such as automobile, electronic, and optical device manufacturing [1]. For example, polymer surfaces with metal layers can be used as surface decorations. This provides an opportunity to replace metal material by using lightweight polymer materials, consequently saving costs in terms of energy and transportation [2]. Printed circuit boards are manufactured from copper-coated polymers by removing an unwanted part of the surface metal layer; a smooth copper layer firmly combined with a polymer substrate is one of the key processes for the application [3].

Current metallization technologies that are generally applied in industries include electro-chemical deposition, thermal spraying, and physical vapor deposition (PVD). Electro-chemical deposition is a method using a substrate as an electrode, depositing a target metal layer with ions from an electrolyte by electro-chemical reactions [4]; it has the advantage of being cost-effective. Therefore, this technology is widely used in industrial manufacturing. However, a limitation exists, as polymers are generally non-conductive. Polymer substrates have to undergo pretreatment before any electro-chemical reaction is carried out. The pretreatment usually includes surface degreasing, chemical etching, surface sensitization and activation, etc. [5]. The purpose of such procedures is to generate a conductive surface on polymer substrates, in turn enabling a self-catalytic reaction thereafter. Sulfuric and chromic acid are widely used in the chemical etching process mentioned to create three-dimensional pores in the micron scale; these chemicals are not environmentally friendly. The required selection of polymer substrates also faces limitations, as most polymers have the advantage of being anti-eroding. In this regard, polymers with double bonds in their molecular structure, such as polycarbonate, acrylonitrile-butadiene-styrene (ABS) resin, are the main choice, as the double bonds can be oxidized by the strong oxidative acids used during the pretreatment processes.

Thermal spraying is a material deposition process whereby a heat source is used to increase the temperature of feed stock particles that are accelerated in a fluid stream through a spray nozzle or torch for consolidation upon impact on a substrate [6]. This technology is suitable to fabricate thick metallic coatings over 100 μm at a relatively high deposition rate [7,8], which can provide improved wear and abrasion resistance, corrosion resistance, and oxidation resistance to the substrates. According to the primary energy source for feed stock particle heating and acceleration, thermal spraying could be classified into cold spraying, flame spraying, electric arc wire spraying, and plasma spraying; the initial gas stream temperature could easily be above 1000 °C [6,9]. In this regard, the choice of polymer substrates is usually limited to heat-resistant or thermoset polymers, such as polytetrafluoroethylene or polyimide [10,11].

When compared to the mentioned polymer metallization technologies, PVD has the advantage of being environmentally friendly, giving more choices for substrate selection due to the relatively lower processing temperatures required. Various PVD technologies, such as magnetron sputtering and arc evaporation techniques, have been used for polymer metallization [12]. Arc evaporation can produce a higher ionization of metallic species and can provide a higher deposition rate, whereas micro-droplets generated during the metal evaporation process results in the surface roughness of metal coatings. Magnetron sputtering presents many advantages, including cost efficiency, scalability, low temperature process, coating uniformity, and surface smoothness. Many PVD coatings have been deposited on polymer successfully by using magnetron sputtering [13,14,15]. However, the poor interfacial adhesion of metallic coatings on polymer substrates due to a low ionization degree of sputtering metal atoms remains the critical problem of magnetron sputtering. Although high-power impulse magnetron sputtering (HiPIMS) can provide a higher ionization of sputtered species (thus refining columnar structures and improving coating toughness [16]), the relatively higher cost of power in using HiPIMS and the lower growth rate of coatings, compared with that of conventional direct current (DC) sputtering, has hampered the widespread use of HiPIMS for such applications.

Various gas (such as Ar, O_2_, and N_2_) plasma pre-treatments have been widely used to improve the interfacial adhesion of sputtered metallic coatings on polymer substrates [17,18]. It is reported that gas plasma pre-treatment could effectively enhance the adhesive strength of the sputtered metallic coatings through the creation of a large density of new adsorption sites, resulting in a larger contact area and incorporation of chemically active groups that lead to increased interaction between the metal and the polymer [17]. Metal ion implantation is usually used to modify the functional characteristics (such as optical, electrical, and biological properties of polymers), whereas the interfacial adhesion due to metal ion implantation is rarely involved [19,20].

In the present work, Cr ion bombardment pretreatment is used to improve the adhesion of sputtered Cu coatings as deposited on ABS substrates. Cr, which has a relatively higher melting point (approx. 1907 °C), was used to prepare an ion flux via arc evaporation decreasing micro-droplets. The influence of the Cr ion energy, as adjusted through a variation in the bias voltage and treatment duration, on the growth structure and properties of Cu coatings was investigated. In particular, the effects of the Cr ion bombardment on the interface structure and the adhesion of the Cu coatings on the ABS substrate were studied. The relationship of the interface structure and adhesive strength is discussed systematically. Experimental results indicate that Cr ion bombardment pretreatment with optimizing of the bias voltage and duration significantly improves the adhesion of sputtered Cu coatings on ABS. Cu coatings with sufficient adhesion strength on ABS processed by DC megnetron sputtering could be an environmentally friendly alternative to current applications, such as automobile interior/exterior manufacturing and circuit board printing.

## 2. Materials and Methods

Surface metallization was carried out for the ABS substrates by using a hybrid process involving magnetron sputtering and arc evaporation. ABS substrates were cleaned in an ultrasonic bath in ethanol for 10 min and then dried in N_2_ streams before putting into a vacuum chamber. The base pressure was reduced to a vacuum of 2 × 10^−3^ Pa without heating. Prior to deposition, the DC glow discharge with a bias voltage of −600 V was used to etch the substrates for 30 s. Subsequently, Cr ions were applied to bombard the ABS substrate surface by cathodic arc evaporation of Cr target (purity 99.95%) in a pure Ar atmosphere (Ar pressure was kept around 0.5 Pa). The cathodic arc current was set at approximately 60 A. Bias voltages ranging from −400 V to −1000 V were applied on the substrate to indirectly adjust the energy of Cr ions. Various treatment durations of the Cr ion bombardment, ranging from 30 s to 5 min, were used. Cu coating was prepared on ABS substrates after Cr ion pretreatment by DC magnetron sputtering using pure Ar to sputter Cu target (purity 99.99%). The distance of the target to the substrate was set to approximately 30 cm to avoid thermal effects. During the deposition process, the deposition pressure was maintained at around 0.3 Pa. The substrate holder rotation speed was set at 2.0 rpm. The sputtering DC power was set at approximately 800 W. The bias voltage was set at −100 V and the deposition duration was 1.5 h.

The surface topography and cross-sectional images of the coatings were observed by scanning electron microscopy (SEM, Hitachi S-4800, Tokyo, Japan). The Cu/ABS samples were cooled by liquid nitrogen first and then segmented for cross-sectional SEM. Transmission Fourier transform infrared spectroscopy (FTIR) (Nicolet iS10, Thermo Fisher Scientific Inc., Waltham, MA, USA) was used to detect the chemical structure changes of the ABS substrate after Cr ion bombardment. The collection parameters were set as follows: resolution 4 cm^−1^, the wave number range was from 4000 cm^−1^ to 600 cm^−1^, 32 scans were performed and averaged per spectrum to improve the signal-to-noise ratio. The adhesion strength of the Cu coatings on ABS substrates was tested by a micro-Scratch Tester (Revetest scratch tester, CSM, Switzerland), using Rockwell C diamond styli with radius 200 μm. A normal load range of 1 to 10 N, scratch length of 3 mm, and scratch speed 6 mm/min were used in the experiments. Post-test characterization of the scratch-tested samples was performed through observations under optical microscopy. The surface resistance of the coating was measured by using a four-probe resistance meter (RTS-9, Guangzhou Four Probe Technology Co., Ltd., Guangzhou, China).

## 3. Results and Discussion

**Figure 1:**
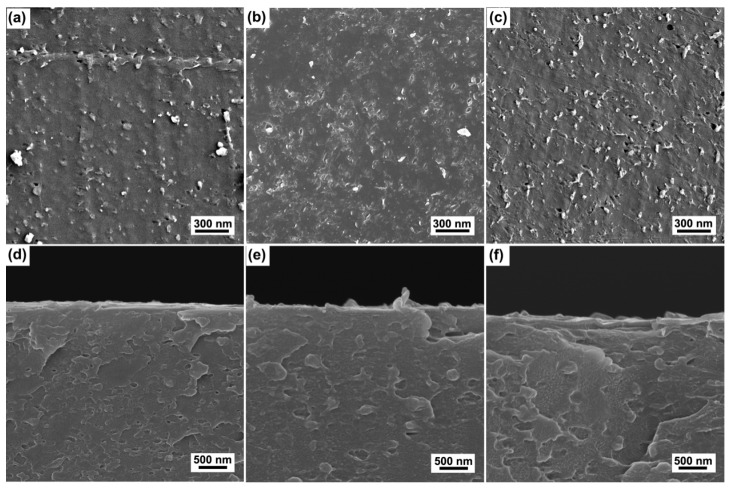
Typical surface (**a**–**c**) and corresponding cross-sectional (**d**–**f**) SEM images of the raw ABS (**a**,**d**) and the ABS substrates treated by Cr ions for 30 s (**b**,**e**) and 3 min (**c**,**f**) with bias voltage of −400 V.

Figure 1 shows the surface and corresponding cross-sectional SEM images of the raw ABS and the ABS substrates treated by Cr ions for 30 s and 3 min with a bias voltage of −400 V. It can be seen that raw ABS (Figure 1a) shows a rough surface with domes. For the sample treated by Cr ions for 1 min (Figure 1b), the surface looks smooth without distinct domes. The corresponding cross-sectional SEM image (Figure 1e) reveals that a very thin layer with a thickness of approximately 30 nm was formed on the sample surface. It is clear that the thin layer was induced by the Cr ion bombardment. The thickness of the Cr layer increases to about 60 nm as the duration increases to 3 min (Figure 1f). It is noted that there are numerous chip-like entities heterogeneously embedded in the surface of the ABS substrate (Figure 1c).

**Figure 2:**
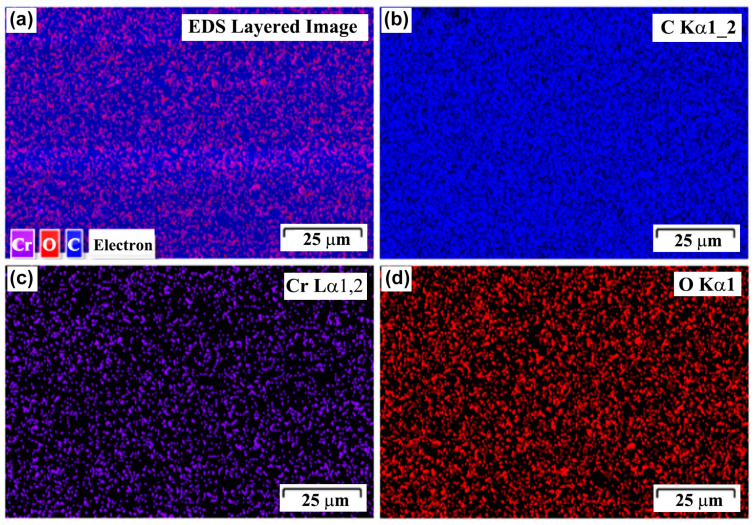
EDS mapping at the ABS substrate treated by Cr ion bombardment for 3 min: (**a**) EDS layered image, (**b**) C, (**c**) Cr, and (**d**) O.

In order to confirm the surface composition of the treated ABS, EDS mapping was performed on the ABS substrate treated by Cr ions for a duration of 3 min, as shown in Figure 2. The surface compositions of the sample reveal the presence of C, Cr (due to Cr ion bombardment), and O (Figure 2a). C (Figure 2b) is found within a homogeneous distribution on the surface, while Cr (Figure 2c) is found in a heterogeneous fashion, in agreement with the corresponding surface SEM image in Figure 1c. Cr is present within chip-like entities embedded in the ABS surface. It can also be confirmed that the thin layers (in Figure 1f) are mainly composed of C rather than Cr. It is reported that the polymer substrates are subject to degeneration under ion bombardment [21]. The formation of the thin layers might be attributed to a degenerative attrition of the ABS surface due to Cr ion bombardment. Similar phenomena were also reported in other literature, where high ion fluence and prolonged treatment induces the formation of an interfacial layer (i.e., amorphous carbon/disordered material) [17,19,22]. O signals could be from the small amount of plasticizers present in the ABS composition.

**Figure 3:**
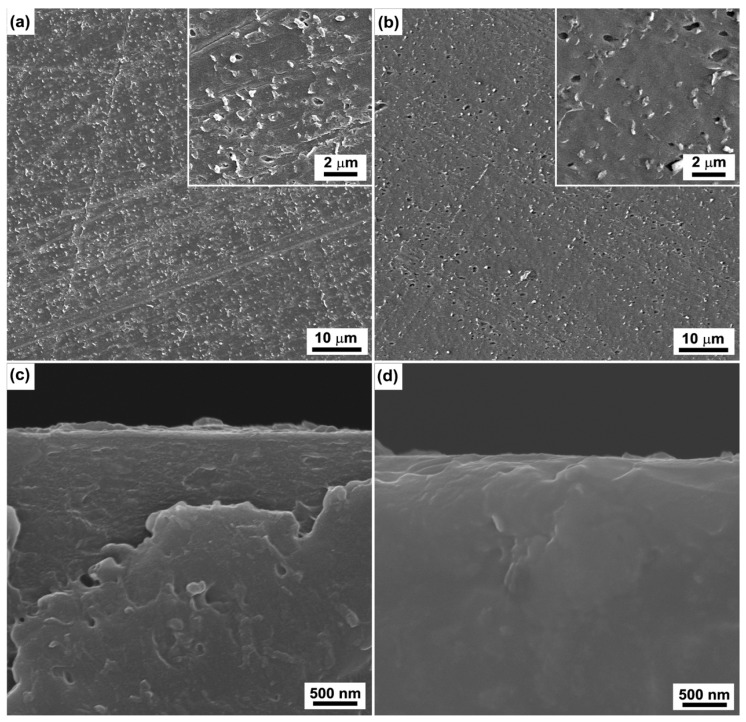
Typical surface SEM images of the ABS substrates treated by Cr ions with bias voltages of −600 V (**a**) and −1000 V (**b**) for 30 s. (**c**,**d**) are the corresponding cross-sectional images of (**a**,**b**), respectively.

Figure 3 presents the surface and corresponding cross-sectional SEM images of the ABS substrates treated by Cr ions with bias voltages of −600 V and −1000 V for 30 s. It can be seen that the Cr particle (chip-like entity) density on the ABS surface bombarded with bias voltage of −600 V is higher than that on the ABS surface bombarded with bias voltage of −400 V. As the bias voltage increases to −1000 V, however, the density of the chip-like entities decreases significantly and the sample surface becomes smooth. It is also worth noting that numerous micro holes now appear on the surface. The formation of the micro holes was associated with thermally induced annihilation of defects, which are common occurrence in polymers treated by high-energy ion bombardment [22,23]. A higher bias voltage (i.e., −600 V as compared with −1000 V) is beneficial for attracting more Cr ions to a substrate. In addition, high-bias voltage would improve the energy of the Cr ions, causing the Cr ions to implant deeply in the ABS surface and remove the chips that have weak adhesion with substrates. However, a bombardment of Cr ions with high energy (−1000 V) would damage the surface, resulting in the formation of the mentioned micro holes.

**Figure 4:**
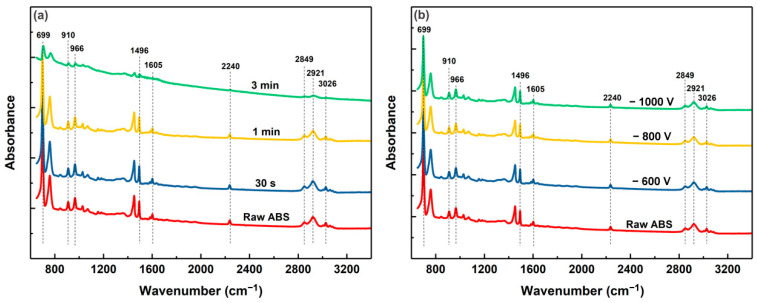
FTIR spectra of the raw ABS and the ABS substrates treated by Cr ions with different duration at bias voltage of 400 V (**a**) and by Cr ions with different bias voltages for 30 s (**b**).

Figure 4 shows FTIR spectra of the raw ABS and the ABS substrates treated by Cr ions with different duration at bias voltage of −400 V and by Cr ions with different bias voltages for 30 s. The typical FTIR spectrum of the raw ABS reveals that the absorption peaks in the range of 3200–3000 cm^−1^ and 3000–2800 cm^−1^ correspond to the stretching vibrations of the aromatic and aliphatic C-H in the ABS, respectively [24]. The peak around 2240 cm^−1^ can be assigned for the C≡N bond, which is the characteristic absorption of the acrylonitrile unit. The absorption around 1605 cm^−1^ represents the stretching vibration of the C=C double bond from the butadiene units, while the peak around 1496 cm^−1^ represents the stretching vibration of the aromatic ring from the styrene unit. The peaks around 966 cm^−1^ and 910 cm^−1^ are ascribed to the deformation of C-H for the hydrogen atoms attached to the alkenic carbons in the 1, 4 butadiene units and the 1, 2 butadiene units, respectively. The absorption peak at 699 cm^−1^ is due to out-of-plane C-H bending of mononuclear aromatic hydrocarbon [25].

It can be seen that the intensity of all the characteristic peaks in the FTIR spectra decrease with the increasing treatment duration of the Cr ion bombardment, as shown in Figure 4a. This indicates that the chemical bonds of the ABS surface indeed deteriorate with Cr ion bombardment. However, with the same treatment duration of 30 s of the Cr ion bombardment, the FTIR spectra of the samples show little change with increasing bias voltage (see Figure 4b), implying that the bombardment energy of the Cr ions has little influence on the chemical bonds of the ABS. It is believed that heat accumulation due to the continuous bombardment by Cr ions may play a major role in the surface deterioration of the ABS substrates.

**Figure 5:**
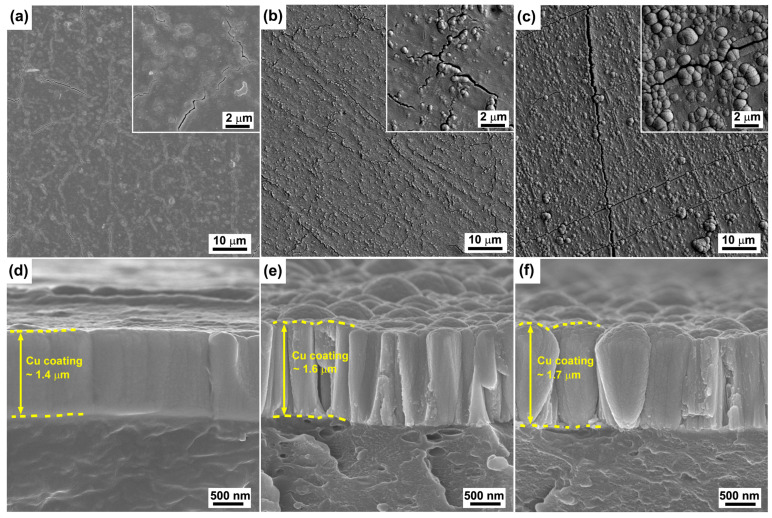
Typical surface topographies of the Cu coatings deposited on the raw ABS (**a**) and the ABS treated by Cr ions for 30 s (**b**) and 3 min (**c**) with bias voltage of −400 V. (**d**–**f**) are corresponding cross-sectional SEM images of (**a**–**c**), respectively.

Cu coatings were deposited on the ABS substrates after treatment with Cr ion bombardment. Figure 5 shows the surface and corresponding cross-sectional SEM images of the Cu coatings deposited on the raw ABS and also the ABS treated by Cr ion bombardment for both 30 s and 3 min at a bias voltage of −400 V. It can be seen that the Cu coating on the raw ABS substrate appears in a smooth topography, except for the presence of some cracks (Figure 5a). The formation of these cracks is mainly attributed to the effects of thermal stress, which often occur during polymer metallization by PVD processes [26]. The corresponding cross-sectional SEM image (Figure 5d) shows a dense and smooth structure. The thickness of the Cu coating is approximately 1.4 μm. For the sample that experienced Cr ion bombardment for 30 s (Figure 5b), the Cu coating shows a granular structure and coarse surface. The corresponding cross-sectional image illustrates the formation of a typical columnar crystal. The appearance of the granular structure on the coating surface is attributed to the caps of the columnar crystals. It should be noted that the columnar size shows a remarkable increase as the duration of Cr ion bombardment increases to 3 min (Figure 5c). In addition, typical cone-like growth structures are observed in the cross-sectional image of the sample (Figure 5f). Furthermore the thickness of the coating increased (~1.7 μm), which might be attributed to the high growth rate of the cone-like structure [27].

**Figure 6:**
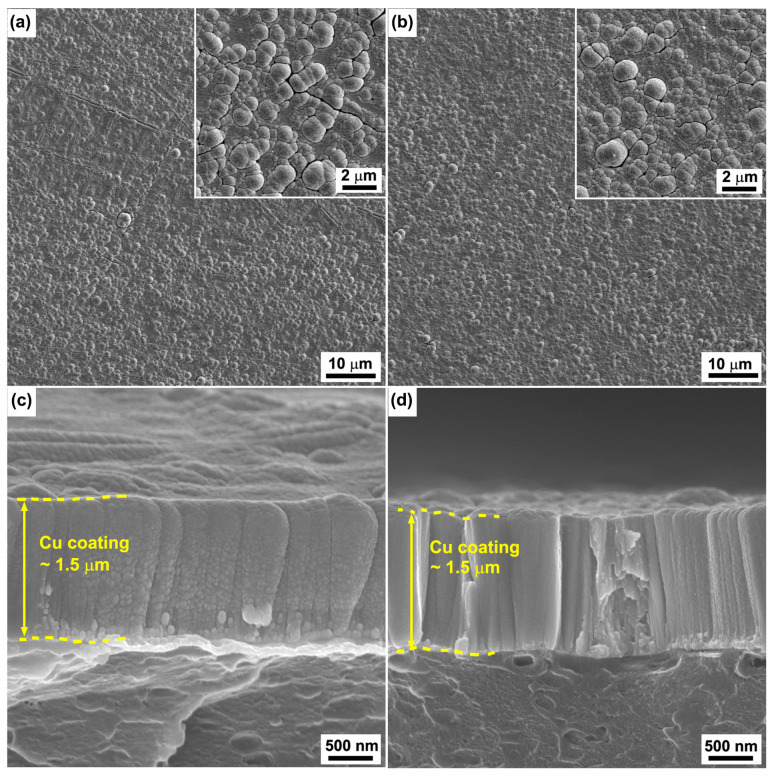
Typical surface SEM images of the Cu coatings deposited on the ABS substrates treated by Cr ions with bias voltages of −600 V (**a**) and −1000 V (**b**) for 30 s. (**c**,**d**) are the corresponding cross-sectional images of (**a**,**b**), respectively.

Figure 6 shows the surface and corresponding cross-sectional SEM images of the Cu coatings deposited on the ABS treated by Cr ions with bias voltages of −600 V and −1000 V for 30 s. It can be seen that the Cu coatings show smooth surfaces (Figure 6a,b) and dense columnar structures. Based on the observations shown in Figure 3, increasing the bias voltage would increase the number of Cr particles and, accordingly, increase the nucleation sites, causing the grain refinement of the Cu coatings. It is worth noting that the larger cone-like grains observed when the duration of the Cr ions increased to 3 min were not observed in the samples, despite the increase of the nucleation sites.

The growth structure of the Cu coatings is expected to be related to the nucleation growth of the Cu coatings. For the raw ABS surface, because the nucleation rate of Cu is similar for the entire surface, the Cu coating on the raw ABS substrate exhibits a smooth and dense structure. For the ABS substrates treated by Cr ions, the Cr particles embedded in the ABS surface could provide the nucleation sites of the Cu coating. Accordingly, the Cu grain nucleated at the Cr particles has the opportunity to grow faster and become bigger, resulting in the formation of coarser, columnar crystals. In addition, the surface chemical state would also have contributed to the growth structure of the Cu coatings. The FTIR spectrum of Figure 4b indicates that the chemical bonds of the ABS surface also remained unchanged as the bias voltage increased. Accordingly, the nucleation rate of the Cu coatings on the ABS treated with high-bias voltage is similar to that of the Cu coatings on the raw ABS. In addition, the Cr particles provide the nucleation sites of the Cu coatings. As a result, the Cu coatings exhibit hybrid structures comprised of dense columnar structure with embedded coarse columnar crystal. For the ABS treated with the duration of 3 min, a thick degeneration layer was formed on the surface, as shown by the results of SEM (Figure 1g) and FTIR (Figure 4a). The degenerated layer may have prevented the nucleation of the Cu coatings and promoted the migration of Cu, resulting in the formation of the larger cone-like columnar structure.

**Figure 7:**
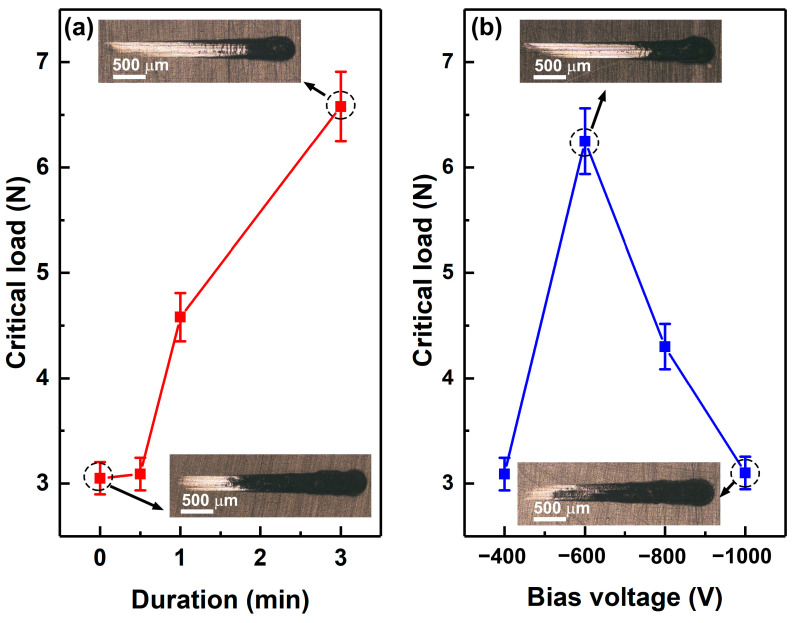
Critical loads of the Cu coatings deposited on the raw ABS and the ABS treated by Cr ions with different durations (**a**) and bias voltages (**b**).

Figure 7 shows the critical loads obtained from scratch tests of the Cu coatings deposited on the raw ABS and the ABS treated by Cr ions with different durations and bias voltages. The critical load when the scratch begins to appear with a coating exfoliation is usually used to evaluate the coating-substrate bonding strength. The insets in Figure 7a show the typical scratch morphologies of the Cu coated on raw ABS and on the ABS treated by Cr ions with bias voltage −400 V for 3 min. The insets in Figure 7b show the typical scratch morphologies of the Cu-coated ABS substrates treated by Cr ions with bias voltages of −600 V and −1000 V for 30 s. It can be seen that the Cu coating deposited on raw ABS substrate shows a critical load of about 3 N. The critical load of the Cu coatings increases with the increasing duration of bombardment, indicating that Cr ion bombardment significantly improves the adhesion of Cu coatings on ABS. It is believed that the Cr particles embedded in the ABS substrates interlock with the Cu coatings and enhance coating adhesion. Increasing the treatment duration of Cr ions increases the embedded Cr particles and, thus, improves the adhesive strength of the Cu coating.

It should be noted that the critical load of the Cu coatings increases first and then decreases as the bias voltage increases, and the maximum of the critical load is observed at the bias voltage of −600 V (see Figure 7b). On one hand, increasing the bias voltage attracts more Cr ions to the substrates and, thus, increases the number of Cr particles. On the other hand, the Cr ions accelerated by high-bias voltage implant deeply into the ABS substrate and greatly enhance the interlocking of the coating and the substrate. However, the amorphous carbon-rich phase was formed on the ABS surface due to Cr bombardment as the bias voltage increases to a certain level. The amorphous carbon phase is expected to be an interfacial layer, which causes the reduction of the adhesive strength between the Cu coating and the ABS substrate [17].

## 4. Conclusions

Cu coating was deposited on ABS substrates by magnetron sputtering with the Cu target. The ABS substrates were pretreated by Cr ion bombardment via cathode vacuum arc evaporation of Cr target. The influence of the bias voltage and the treatment duration of Cr ion bombardment on the growth structure and adhesion of the Cu coatings was studied. It is found that the Cr ion bombardment led to the formation of Cr particles embedding in the surface. The Cr particles interlocked with the Cu coatings and ABS substrate and significantly improved the coating adhesion. In addition, the Cr particles acted as the nucleation sites of the Cu coatings and facilitated the growth of columnar crystals. The coating adhesion increased as the treatment duration increased. However, continuous bombardment resulted in a degenerated layer on the ABS surface. The degenerated layer is expected to be unfavorable for the nucleation of the Cu coating. As a result, the Cu coating deposited on the ABS substrate treated with a long duration of Cr ion bombardment showed larger cone-like columnar structures. Increasing the bias voltage increased the Cr particles without causing the degeneration of the ABS surface. Accordingly, the Cu coating deposited on the ABS substrate treated by Cr ion with high-bias voltage and short duration showed a dense and smooth growth structure. However, Cr ion bombardment with high-bias voltage caused the emergence of an interfacial layer (amorphous carbon-rich phase) in the ABS surface, resulting in the reduction of the coating adhesion. It is believed that the pretreatment of Cr ion bombardment with an appropriate bias voltage and duration aided in preparing Cu coatings on the ABS substrate. By strengthening the adhesion and refining the micro-structure of the Cu deposited on the ABS substrate, DC magnetron sputtering could be a more viable technology to add value to applications such as conductive surfaces and lightweight decorative components.

## Data Availability

The data used to support the findings of this study are available from the corresponding author upon request.
